# Oral theophylline corrects sinus node dysfunction in acute on chronic lithium toxicity: case report and systematic review of lithium-induced Sinus node dysfunction

**DOI:** 10.3389/fcvm.2024.1412376

**Published:** 2024-08-29

**Authors:** J. Curran Henson, Lauren Morehead, Joshua Hagood, Niroshi Wijewardane, Hakan Paydak

**Affiliations:** ^1^ Department of Internal Medicine, University of Arkansas for Medical Sciences, Little Rock, AR, United States; ^2^ Division of Cardiology, Department of Internal Medicine, College of Medicine, University of Arkansas for Medical Sciences, Little Rock, AR, United States; ^3^ Division of Cardiology, Department of Internal Medicine, University of Arkansas for Medical Sciences, Little Rock, AR, United States

**Keywords:** lithium, lithium toxicity, sinus bradycardia, sick sinus syndrome, sinus node dysfunction, arrythmia, temporary pacing, hemodialysis

## Abstract

**Introduction:**

Lithium is a well-known agent to cause systemic toxicity with its narrow therapeutic window. Toxic cardiac effects are known but seldomly reported and can manifest as sinus node dysfunction (SND) ranging from delayed conduction to sinus arrest with the potential to induce asystole. Theophylline a positive chronotropic agent has been previously used for correction of sinus node dysfunction but never been utilized for the correction of lithium-induced SND. We report the first successful use of Theophylline for rate/rhythm correction of SND in a patient presenting with acute lithium toxicity along with a review summarizing the clinical features of all published literature regarding lithium-induced SND.

**Methods:**

Case report and systematic review of the literature are presented. Three independent scientific databases were queried for reports of lithium-induced SND. A clinical compendium was then generated detailing associated clinical data and descriptive statistics were performed.

**Results:**

1,117 reports were initially retrieved with full-text review yielding a cohort of 49 unique, independent studies. (61.4%) of patients presented with a supratherapeutic lithium level, 12 (21.1%) were normotherapeutic, and 11 (19.3%) were subtherapeutic. EKG findings varied but most commonly described sinus node dysfunction with a variable degree of sinoatrial block with sinus bradycardia (54.39%) and sinus arrest (29.82%) predominating. Twelve patients (21.1%) required inotrope or vasopressor support. 10 (17.5%) of patient required temporary pacing while 7 (12.3%) required permanent pacemaker implantation. In the majority of cases no significant permanent sequelae were reported as 50 (87.7%) patients recovered, 2 (3.5%) patients had persistent sinus node dysfunction, and 2 (3.5%) patients expired as a result of acute lithium toxicity.

**Discussion & conclusion:**

In this review we report the most up-to-date and comprehensive clinical compendium of lithium-associated sinus node dysfunction along with describing a novel treatment methodology to rapidly correct lithium-induced cardiac toxicity in a patient with long-standing bipolar disorder on chronic lithium treatment. We have reviewed the available literature and provide a comprehensive summary detailing symptomatology of presentation, treatments utilized, electrocardiographic findings and patient prognoses. We have concluded that under the presumptive conditions that transient sinus node dysfunction will resolve with elimination of toxic concentrations of lithium, temporary chronotropic support provided by theophylline administration would be preferable to more invasive measures such as hemodialysis, temporary pacing, or implantation of a permanent pacemaker.

## Introduction

Sinus node dysfunction (SND), also known as sick sinus syndrome (SSS), refers to a distinctive set of arrhythmogenic bradycardias with particular electrocardiographic patterns. Clinically, sinus node dysfunction can manifest on electrocardiogram (EKG) as either a regular or irregular bradycardia with heart rate <60 beats per minute (bpm) and present with symptoms of fatigue, dizziness, chronotropic incompetence, and syncope (1). Diagnosis requires a combination of clinical symptoms and EKG findings (2, 3). SND or sinoatrial block is similar to atrioventricular block (AV) in that it has varying classifications ranging in degree of severity, from Type I manifesting as delayed depolarization and conduction to Type III resulting in long sinus pauses maintained by junctional or idioventricular escape rhythms, or leading to complete sinus arrest. Pathophysiological disruptions of this intracardiac sinoatrial circuit can be caused by a multiplicity of pathologies causing direct damage to the sinus node ranging from acute myocardial infarction, embolization of the sinoatrial node artery, cardiomyopathy, diabetes, hypertension, metabolic derangements, to polypharmacy (2–6). Lithium, a common agent utilized in the treatment of chronic psychiatric conditions, including bipolar and schizoaffective disorders, is a known but seldomly reported pharmacological mediator of sinus node disruption. Lithium has been reported to disrupt intrinsic sinus node pacemaker activity through interaction with pacemaker (HCN) channels (7) and/or the sodium-calcium exchanger (8) thereby impairing pacemaker activity of the SA nodal cells which govern the complex physiological mechanism of cardiac pacing (8–10). Lithium, with its therapeutic window [0.8–1.2 mmol/L] has been shown to not-only induce sinus node dysfunction at supratherapeutic concentrations but, in some reports, at both therapeutic and subtherapeutic levels. Various treatment modalities have been utilized in acute lithium toxicity, ranging from simple observation to emergent hemodialysis, temporary pacing, and permanent pacemaker implantation, but the use of positive chronotropic agents for the treatment of acute lithium-induced SND has never been reported.

Theophylline, a methylxanthine known to have positive chronotropic effects, has been suggested for off-label use for patients with sinus node dysfunction or sick sinus syndrome (SSS) (11–13). In a comparative retrospective study evaluating the efficacy of theophylline vs. cilostazol for treatment of SSS in patients who refused pacemaker implantation, theophylline increased heart rate from baseline by 12.0 +/− 16.3 bpm by sphygmomanometry, (*p* < 0.001), by 8.4 bpm +/− 12 by EKG recording (*p* < 0.001), and was shown to be non-inferior to cilostazol, (*p* = 0.338). Symptomatic improvement in chest discomfort, dyspnea, syncope, and palpitations was noted after administration of both medications, with improvement in dizziness being superior in the theophylline group (*p* < 0.003) (11). In another study of patients with SSS, theophylline decreased the frequency of sinus pauses from 256 ± 230 to 23 ± 62 pauses per 24 h and decreased the duration of the longest pauses from 4.7 ± 1.8 s to 2.2 ± 0.97 s after one month of treatment, with disappearance of subjective symptoms associated with cardiac pauses in 16 of 17 patients (12). Lastly, in the treatment of acute COVID-19 induced sinus bradycardia, theophylline has been shown to rapidly improve heart rate, induce reversion to normal sinus rhythm, and cause complete resolution of lightheadedness and dizziness (13). It is with these presuppositions we aim to describe the first reported use of theophylline for rapid correction of lithium-induced SND while simultaneously presenting the most up-to-date and comprehensive clinical compendium of the associated literature.

## Case report

**Figure 1:**
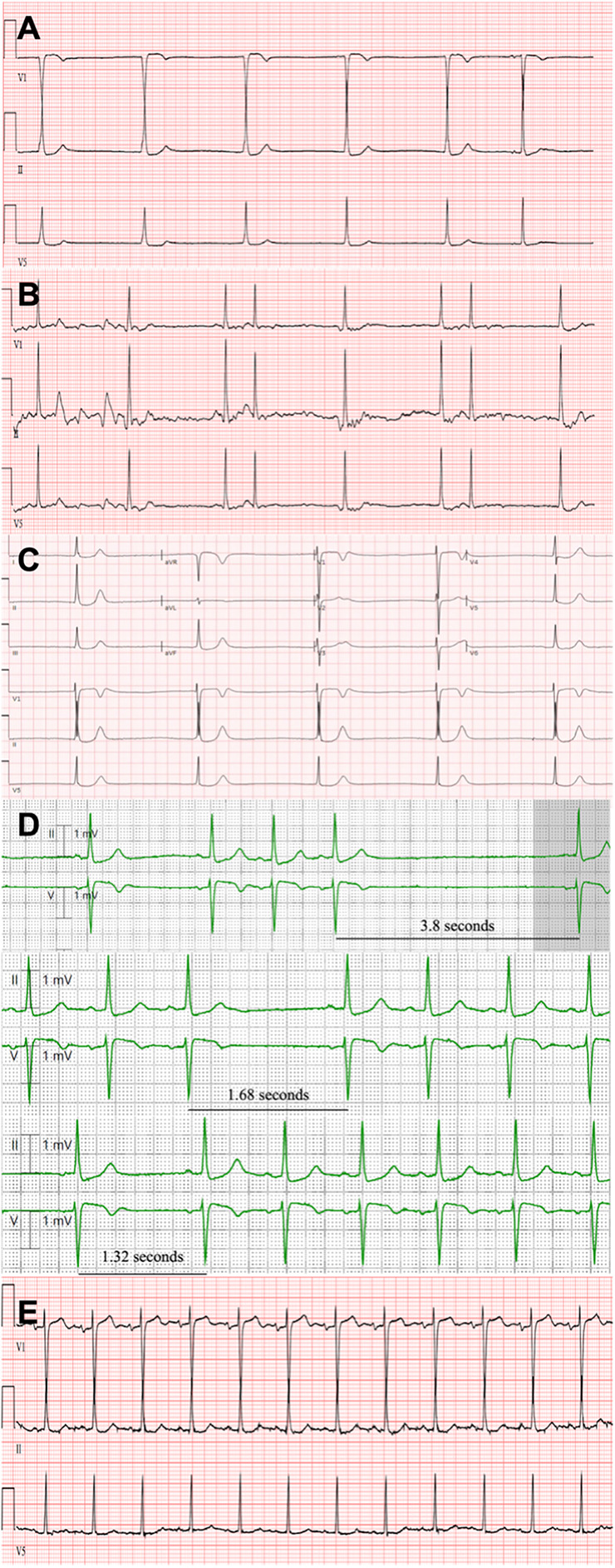
**(A)** Initial EKG at obtained in the emergency department at time of patient presentation with evidence of junctional escape rhythm and noted absence of p-waves indicative of sinoatrial dysfunction in all but the last sinus complex. **(B)** Repeat EKG 30-minutes after administration of atropine showing normal sinus rhythm with emergence of p-waves and bigeminal premature atrial complexes indicative of sinus node and atrial conduction recovery. There is noted lead misplacement of V1 indicated by only positive deflection of QRS complexes not consistent with antecedent and subsequent studies. **(C)** Repeat EKG obtained 12-hours after atropine administration revealing complete dissipation of sinus node recovery with re-emergence junctional escape rhythm and noted absence of p-waves indicative. **(D)** Sequential progression of telemetry-capture approximately 12-hours after initial Theophylline dose revealing progressively shortening sinus pauses ranging from sinus arrest of 3.8 s to a briefer pause of 1.12 s to eventual complete resolution of SND with return to normal sinus rhythm. **(E)** EKG obtained approximately 24 h after theophylline administration with evidence of sustained normal sinus rhythm and regular p-waves with normal sinoatrial conduction pattern.

In this report we describe the first successful use of theophylline to correct lithium-induced sinus node dysfunction in a patient on long-term lithium maintenance therapy. We present the case of a 73-year-old female with long-standing comorbid schizophrenia and bipolar disorder who presented to our emergency department in November of 2023 with complaints of confusion, progressive fatigue and global weakness that had developed over the prior few months, as well as a symmetric fine-motor tremor of the bilateral upper extremities. The patient had a past medical history of insulin-dependent diabetes and hypertension. Home medications included neutral protamine Hagedorn (NPH) insulin 30 units administered twice daily, lisinopril 2.5 mg once daily, risperidone 3 mg once daily, and lithium 300 mg once daily. Her initial vitals were significant for bradycardia with normotensive pressures, a heart ranging from 31 to 56 and systolic blood pressure ranging from 108 to 131. Initial EKG Figure 1(s) obtained upon presentation showed evidence of sinoatrial block with junctional escape rhythm.

She was given 1 mg of atropine IV with response of heart rate to around 60 beats per minute. Initial lithium level was assessed and found to be 2.2 mmol/L [0.6–1.2 mmol/L]. Repeat EKG (Figure 1B) after the administration of atropine exhibited slight recovery of sinus function with emergence of p-waves and bigeminal premature atrial complexes indicative of improvement in atrial conduction.

At the time of presentation, the patient also exhibited an acute kidney injury (AKI) with creatinine of 2.6 mg/dl due to poor oral intake. TSH was checked and found to be within normal limits with a value of 3.77 uIu/ml. Other significant lab findings were a mild leukocytosis of 11.22 K/ml, and hyperkalemia of 6.6 mmol/L for which she was treated with calcium gluconate for myocardial stabilization. Multiple teams, including psychiatry, nephrology, and toxicology, were consulted for coordination of care in the setting of this complex presentation. The patient described in this case did not exhibit capacity during initial presentation and the patient's spouse desired to avoid invasive measures at all possible costs including escalation to a higher level of care. Given these limitations it was determined that the best course of action would be to proceed with judicious fluid resuscitation while monitoring continued response to atropine while the patient remained hemodynamically stable. She was admitted to the cardiology service and continued on normal saline at a rate of 150–200 (ml/hr) along with close surveillance and serial monitoring of lithium level every two hours. Pending decompensation, the patient's spouse was agreeable for transfer to the medical intensive care unit (ICU) for initiation of renal replacement therapy.

Although it appeared that atropine provided satisfactory heart rate recovery for multiple hours, sustained sinus node recovery was not observed. Approximately 12 h after atropine administration, the patient suffered deteriorating sinus node function with redevelopment of junctional bradycardia with a ventricular rate of 31 bpm (Figure 1C). Given that the patient remained hemodynamically stable albeit severe sinus bradycardia, it was decided that an alternate therapy would be best tried prior to escalation of care and initiation of chronotropic agent infusion which would require ICU transfer. Theophylline 300 mg was trialed. 12-hours subsequent to theophylline dosing, telemetry-capture (Figure 1D) revealed progressively shortening sinus pauses from sinus arrest of 3.8 s to a briefer pause of 1.32 s to eventual complete resolution of SND with return to normal sinus rhythm. The following morning, a repeat EKG (Figure 1E) was obtained that confirmed the patient had reverted to normal sinus rhythm, with a heart rate of 74 bpm—an increase of 43 bpm from the prior EKG.

The patient was maintained on telemetry throughout the remainder of her admission and did not experience anymore recorded telemetric events that evidenced sinus bradycardia or SND. She was monitored for an additional 24-hrs and given a second dose of Theophylline 300 mg the following morning. She was subsequently discharged with antihypertensive dose adjustment for improved blood pressure control and instructions for appropriate outpatient psychiatric follow-up for initiation of an alternate psychiatric medication regimen. Her Lithium was held at discharge.

## Systematic review methods

**Figure 2:**
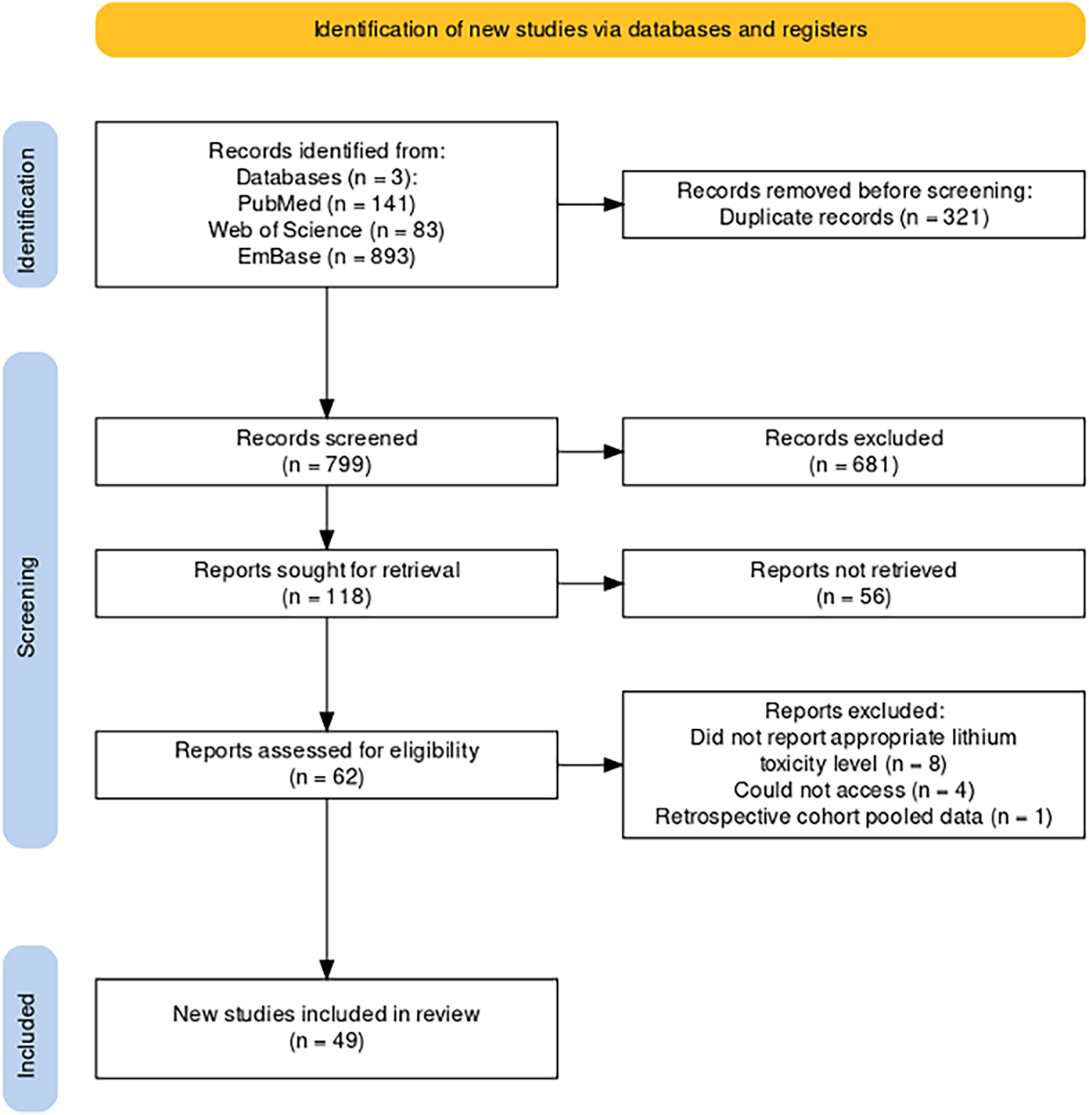
PRISMA diagram (14) of workflow for selection and retrieval of articles included in our literature review utilizing search engines pubMed, Web of science, and embase.

A systematic review was conducted to summarize and gain a better understanding of the treatment methodologies that currently exist as standard of care for lithium induced sinus node dysfunction and to explore in this context the associated patient outcomes (Figure 2). Query was performed utilizing three comprehensive scientific research databases (Pubmed, Embase, and Web of Science) to assess lithium induced sinus node dysfunction and to catalog the treatments and patient outcomes available in clinical literature using the search terms [(“Lithium”) AND (‘Sinus Node Dysfunction’ OR ‘Sick Sinus Syndrome’ OR ‘Sinoatrial Block’)] which resulted in 1,117 reports. This query was then deduplicated using reference software Zotero resulting in 799 unique independent reports. From this cohort, initial abstract screen was performed with further exclusion of 681 reports resulting in 118 records that were sought for retrieval. Subsequently, inclusion and exclusion criteria were applied thereby resulting in further exclusion of 56 articles. Articles reporting sinus node dysfunction or lithium-induced bradyarrhythmia as a result of any form of toxicity (acute vs. acute on chronic vs. chronic) were included in initial abstract screen. Secondary screening consisted of evaluation for records with documented electrocardiographic evidence of sinus node dysfunction, or sinoatrial block along with a documented recording of initial lithium level at presentation (both subtherapeutic, therapeutic, and supratherapeutic). On final abstract screen the following inclusion criteria were applied: retrospective or prospective studies, case reports, case series, randomized controlled trials, age >18 years old. Exclusion criteria included lithium-associated other tachy- or bradyarrhythmia (i.e., ventricular arrythmias or AV block), meta-analyses, review articles, animal studies, <18 years old, articles not in English. Lastly, 62 articles then underwent full-text review for resulting in further exclusion of 13 studies for the following reasons: *n* = 8, did not report appropriate lithium toxicity level or have electrocardiographic evidence of sinus node dysfunction, *n* = 4, article unavailable to be retrieved, *n* = 1 retrospective cohort with pooled data and inappropriate reporting of outcomes. This resulted in a final cohort of 49 unique, independent studies included in our review (Table 1).

**Table 1:** Data compendium compiled from literature review of lithium-induced sinus node dysfunction.

Author(s)	Patient age	Classification of lithium toxicity	Duration of lithium treatment	Initial lithium level	Repeat lithium level	Home meds (excluding lithium)	Presenting symptoms	EKG findings	Treatment	Outcomes
Akhigbe et al.	67	Acute on Chronic	n/a	1.8	Not reported	Lisinopril	Syncope	Junctional escape rhythm with retrograde p-waves	Observation	Not reported
Ataallah et al.	42	Acute on Chronic	n/a	0.55	Not reported	Not reported	Dizziness and fatigue	Sinus bradycardia with non-specific t-wave inversions	Observation	Recovered
Ben Fredj et al.	35	Acute	7 days	0.3	Not reported	Valproic acid, haloperidol, lorazepam	Asymptomatic bradycardia	Sinus bradycardia	Observation	Recovered
Bogin et al.	61	Acute on Chronic	n/a	2.57	Not reported	Lisinopril	Dyspnea, confusion, and fatigue	Sinus bradycardia with escape capture bigeminy	Observation, inotropic support	Recovered
Bravo et al.	68	Acute on Chronic	12 years	1.67	0.92	Carbamazepine, pipamperone, zopiclone, mirtazapine, rofecoxcib	Tremor, hypokinesia	Sinus bradycardia, sinus arrest, and intermittent complete av block	Observation, vasopressor support	Recovered
Chen et al.	76	Acute on Chronic	5 years	5	1.3	Losartan, perphenezine	Ataxia, anorexia	Sinus bradycardia with ventricular escape rhythm, non-specific st-t segment changes	Hemodialysis	Recovered
Dahan et al.	61	Chronic	9 years	1.2	0.7	Venlafaxine cr, clonazepam, risperidone,	Global weakness, tremors	Sinus bradycardia with non-specific t-wave inversions	Pacemaker (permanent)	Recovered
Demirtas et al.	82	Acute on Chronic	20 years	1.3	0.02	Quetiapine, ramipril, levodopa/benserazide, ibandronic acid	Weakness, fatigue, di, palpitations	Sinus bradycardia	Pacemaker (temporary)	Recovered
Farag et al.	75	Acute on Chronic	20 years	2.13	Not reported	Not reported	Syncope and collapse	Narrow-complex junctional bradycardia	Observation	Recovered
Goldberger	43	Acute on Chronic	> 1 year	3.64	0.41	Naproxen, amitriptyline, mirtazapine	Hypothermia	Sinus bradycardia and type ii sa block sinoatrial block with an intermittent 2:1 conduction pattern, with non-specific t-wave flattening	Hemodialysis	Recovered
Gupta et al.	55	Acute on Chronic	n/a	2.1	Not reported	Raminpril, aspirin, clopidogrel, carbamazepine	Nausea, vomiting, syncope	Junctional escape rhythm with sinus arrest	Pacemaker (temporary)	Recovered
Hagman et al.	75	Chronic	2 years	0.3	Not reported	Levopromazine, thioridazine, digoxin	Fatigue, repeated syncope	Sinus bradycardia, sinus arrest, and junctional escape rhythm, non-specific t-wave changes, st segment abnormalities	Observation	Recovered
Hagman et al.	63	Chronic	6 years	1.1	Not reported	Chlorpromazine, haloperidol	Repeated syncope	Sinus bradycardia, episodes of asystole without junctional escape rhythm	Observation	Recovered
Hussain et al.	81	Acute on Chronic	n/a	2.7	1.2	None	Syncope and collapse	Sinus bradycardia and pvcs with conversion to atrial fibrillation, t-wave inversion in inferior leads	Inotropic support, pacemaker (temporary)	Recovered
Khalid et al.	68	Acute on Chronic	n/a	2.04	Not reported	Aspirin, enalapril, atorvastatin, haloperidol, lithium, trihexyphenidyl, valbenazine, and levothyroxine	Decreased po intake, dysphagia, lethargy	Sinus bradycardia and junctional escape rhythm with right bundle morphology	Observation	Recovered
Kumar et al.	48	Chronic	20 years	1.16	Not reported	Risperidone	Dizziness and syncope	Sinus bradycardia, sinus arrest, and junctional escape rhythm	Atropine, inotropic support, mechanical ventilation, pacemaker (temporary)	Recovered
Lahmeyer et al.	56	Acute on Chronic	9 years	2.2	Not reported	None	Altered mental status, lethargy, hypotension, unresponsiveness	Sinus bradycardia, diffuse st elevations in inferior and anterior leads, and qt prolongation	Pacemaker (temporary)	Recovered, persistent diabetes insipidus, and dyskinesia
Lai et al.	42	Acute on Chronic	> 2 years	3.86	0.15	Carbamazepine, clonazepam, trazodone, moperone, bethanechol, trihexyphenidyl	Tonic-clonic convulsion, loss of consciousness, diarrhea for 1-week, dizziness, ataxia, tremor, frequent falls, and drowsiness for 3-days	Sinus bradycardia, sinus arrest, and inverted t-waves with prominent u-waves	Hemodialysis	Recovered
Maddala et al.	77	Acute on Chronic	30 years	2.6	0.9	Losartan, metoprolol, levothyroxine	Dizziness, bilateral hand tremor	Junctional rhythm	Pacemaker (temporary)	Recovered
Martinez et al.	69	Acute on Chronic	n/a	2.32	Not reported	Olmesartan-amlodipine, venlafaxine, lamotrigine	Dizziness, asthenia, adynamia, repeated syncope	Second-degree type ii sa block, t-wave flattening	Observation	Not reported
Mehta et al.	54	Acute on Chronic	n/a	3	1.2	Amitriptyline, quetiapine, zolpidem	Confusion, insomnia, agitation	Junctional escape with premature bigeminy complexes, brugada pattern, and non-specific t-wave flattening	Observation	Recovered
Montal-ESCOT et al.	56	Chronic	12 years	0.8	0.1	Not reported	Dizziness, dysmetria, disequilibrium, mild dyspnea	Sinus bradycardia, and depression of t-waves in posterior segments	Atropine, pacemaker (temporary)	Recovered
Musfeldt et al.	61	Acute on Chronic	n/a	1.51	0.54	Not reported	Lightheadedness, dizziness, weakness, fatigue	Sinus bradycardia, and junctional escape rhythm	Observation	Recovered
Nagamine et al.	78	Acute on Chronic	3 months	2.04	0.51	Clonazepam	Repeated syncope	Junctional escape rhythm, and av disassociation	Observation	Recovered
Nakamura et al.	36	Acute	1 week	0.61	Not reported	Olanzapine, paliperidone, eszopiclone	Exacerbation of schizophrenic symptoms	Sinus bradycardia with partial fusion idioventricular escape rhythm	Observation	Recovered
Naranyan et al.	37	Acute on Chronic	n/a	3.7	0.55	Not reported	Unresponsiveness	Sinus bradycardia, junctional escape rhythm, t-wave inversion, qt prolongation	Mechanical ventilation, inotropic support, hemodialysis	Recovered
Numata et al.	39	Chronic	6 years	0.9	Not reported	Not reported	Dizziness, chest discomfort	Sinoatrial block, and sinus arrest	Observation	Recovered
Numata et al.	58	Chronic	2 years	0.7	Not reported	Not reported	Syncope	Sinus bradycardia, and sinus arrest with frequent pvcs	Observation	Recovered
Nykiel et al.	61	Acute on Chronic	n/a	1.51	Not reported	Not reported	Lightheadedness, weakness, fatigue	Sinus bradycardia with junctional escape rhythm	Atropine, inotropic support, pacemaker (chronicity not specified)	Recovered
Oudit et al.	64	Chronic	11 years	0.72	Not reported	Not reported	Presyncope	Sinus arrest with idioventricular escape rhythm	Atropine, observation	Recovered
Palileo et al.	61	Chronic	1 year	0.75	Not reported	Not reported	Repeated syncope	Sinus bradycardia, sinus arrest, and junctional escape rhythm	Pacemaker (permanent)	Recovered, persistent sinus node dysfunction
Pavuluri et al.	45	Acute on Chronic	n/a	3.31	Not reported	Lisinopril, furosemide	Altered mental status, diarrhea, unresponsiveness	Sinus bradycardia with prolonged qrs and prolonged qt followed by pulseless wide-complex bradycardia and asystole	Acls code, sustained low-efficiency dialysis (sled)	Deceased
Rector et al.	60	Chronic	9 months	0.8	Not reported	Diuretic (unspecified), methyldopa	Asymptomatic	Sinus bradycardia, and second-degree type i (wenckebach) sa block	Pacemaker (permanent)	Not reported
Riccioni et al.	74	Acute on Chronic	6 months	2	1.27	Not reported	Confusion, ataxia, syncope	Alternating sinus bradycardia, supraventricular tachycardia, and junctional rhythm	Atropine, vasopressor support, observation	Recovered
Rijal et al.	37	Acute on Chronic	n/a	3.7	1.2	Lamotrigine, lisinopril, levothyroxine, metformin, glipizide	Vomiting, decreased po intake, tremor	Sinus bradycardia with sinus pauses, junctional rhythm with escape-capture bigeminy	Hemodialysis	Recovered
Rodney et al.	59	Acute on Chronic	2 years	1.3	0.1	Not reported	Asymptomatic	Junctional rhythm with ventricular bigeminy	Atropine, observation	Recovered, persistent sinus node dysfunction
Roose et al.	69	Chronic	10 years	1.1	Not reported	Triiodothyronine	Dyspnea on exertion, chronotropic incompetence	Sinus pauses with junctional escape rhythm	Pacemaker (permanent)	Recovered
Roose et al.	75	Acute on Chronic	9 years	1.8	1.5	Not reported	Ataxia, choreiform movements of fingers and hands	Sinus bradycardia with junctional escape rhythm, lbbb, and t-wave inversions	Observation	Recovered
Roose et al.	53	Chronic	3 months	1	Not reported	Not reported	Asymptomatic	Sinus pauses with junctional escape rhythm	Observation	Recovered
Sabharwal et al.	27	Acute	1 day	<0.2	Not reported	Unspecified psychiatric medications	Dizziness with confusion	Sinus bradycardia with right rbbb	Atropine, observation	Recovered
Sarangi et al.	74	Chronic	5 years	0.6	Not reported	Lisinopril, indomethacin, terazosin, furosemide, metoprolol, aspirin, vitamin c	Dizziness	Sinus bradycardia with prolonged pr interval, rbbb, and left anterior fascicular block	Pacemaker (permanent)	Recovered
Shetty et al.	46	Chronic	15 years	0.7	Not reported	Not reported	Dizziness	Sinus arrest with junctional escape rhythm	Observation, pacemaker (temporary)	Recovered
Snipes et al.	60	Acute on Chronic	n/a	3.3	1.6	Atorvastatin, baclofen, carbamazepine, carvedilol, clonazepam, levetiracetam, mirtazapine	Altered mental status, diarrhea	Sinus bradycardia and non-specific interventricular conduction delay	Atropine, glucagon, inotropic support, pacemaker (temporary)	Recovered
Steckler	56	Acute on Chronic	3 years	2.05	1.21	Carbamazepine	Ataxia	Sinus bradycardia, sinus pauses	Observation	Recovered
Steckler	26	Acute on Chronic	n/a	1.5	Not reported	Carbamazepine, perphenazine, propranolol	Syncope	Sinus arrest and junctional bradycardia	Observation, vasopressor support	Recovered
Steckler	53	Acute on Chronic	5 months	1.66	Not reported	Haloperidol, cogentin, carbamazepine	Syncope	Sinus arrest and junctional bradycardia	Observation	Recovered
Steckler	75	Acute on Chronic	n/a	1.32	Not reported	Haloperidol, carbamazepine	Nausea, vomiting	Sinus arrest with junctional escape rhythm	Observation	Recovered
Steckler	40	Chronic	n/a	0.96	Not reported	Not reported	Mania, nausea, vomiting	Sinus bradycardia with prolonged pr interval with junctional and ventricular fusion escape beats, complete heart block	Observation	Recovered
Talati et al.	58	Chronic	n/a	1.1	Not reported	Levothyroxine, glyburide	Generalized weakness, fatigue	Sinus bradycardia with junctional escape rhythm	Atropine, observation	Recovered
Terao et al.	56	Chronic	6 years	0.7	Not reported	Valproic acid, haloperidol, levomepromazine, flunitrazepam	Syncope, mania	Sinus bradycardia with ventricular escape beats, sinus arrest	Observation	Recovered
Tuman et al.	42	Acute on Chronic	8 years	1.8	0.4	Not reported	Syncope	Sinus bradycardia, and a-fib with slow ventricular response	Pacemaker (permanent)	Recovered
Venkatara-thnamma et al.	21	Chronic	n/a	0.8	Not reported	Haloperidol, trihexyphenidyl	Vomiting, respiratory failure	Sinus bradycardia, st segment depression and t-wave inversion in anterior and inferior leads	Mechanical ventilation, inotropic support	Deceased
Weintraub et al.	60	Acute on Chronic	5 years	1.25	Not reported	Not reported	Non-specific chest pain	Sinus bradycardia with non-specific t-wave inversions	Atropine, observation	Not reported
Wellens et al.	56	Chronic	6 years	1	Not reported	Not reported	Syncope, dizziness, fatigue	Irregular sinus rhythm, sinus arrest	Observation	Not reported
White et al.	54	Acute on Chronic	n/a	2.84	Not reported	Atenolol, gabapentin	Lethargy, chest discomfort, presyncope	Sinus arrest with irregular junctional escape rhythm	Atropine, pacemaker (temporary), hemodialysis	Recovered
Woods et al.	70	Acute on Chronic	n/a	2.3	0.6	Glargine, prophylactic heparin	Altered mental status, hypotension	Sinus bradycardia, sinoatrial block, and junctional escape rhythm	Observation, vasopressor support	Recovered
Worthley et al.	52	Acute on Chronic	5 years	3	Not reported	None	Cardiac arrest requiring cardioversion	Sinus bradycardia with non-specific t-wave flattening, prolonged qt interval, frequent ectopic ventricular beats	Observation, repeat cardioversion, vasopressor support, magnesium infusion	Recovered

## Results

The number of individual patients included in our review totaled 57 with a mean age of 57.42 years aging from 21 to 82 years. As a distinct classification scheme for lithium toxicity is not well defined, for the purposes of this study we defined our classification of lithium toxicity as follows; acute toxicity is differentiated as either an intentional overdose or presentation of toxicity upon new initiation of lithium therapy regardless of initial lithium level, acute on chronic refers to patients with toxic symptomatology and supratherapeutic lithium levels that have been previously stable on long-standing lithium treatment, and chronic toxicity is defined as patients with toxic symptomatology with normo- or subtherapeutic lithium levels. Three patients (5.2%) were classified as acute, thirty-five were classified as acute on chronic (61.4%), and nineteen (33.3%) were classified as chronic. Of our included cases, (63%) reported treatment duration. Average treatment duration classified by type of toxicity are as follows: acute—5 days, acute on chronic—7.77 years, and chronic—7.0 years. The initial lithium level at presentation ranged from 0.2–5, with a mean of 1.73, and standard deviation of 1.05 with 32 (61.4%) of patients being supratherapeutic, 12 (21.1%) normotherapeutic, and 11 (19.3%) subtherapeutic. Repeat lithium level was reported in 22 (38.6%) of cases ranging from 0.02–1.6, with a mean of 0.78 and a standard deviation of 0.48. The most common reported symptom was syncope (19.30%), followed by dizziness (12.28%), and fatigue (10.53%). EKG findings varied but most commonly described sinus node dysfunction with a variable degree of sinoatrial block with sinus bradycardia (54.39%) and sinus arrest (29.82%) predominating. Regarding the various treatment regimens utilized, the majority of patients underwent simple observation 22 (38.6%) as compared to 7 (12.3%) who required urgent hemodialysis in the acute setting. Twelve patients (21.1%) required inotrope or vasopressor support. In regard to the use of pacing for symptomatic bradycardia, 10 (17.5%) patients required temporary pacing while 7 (12.3%) required permanent pacemaker implantation. In the majority of cases no significant permanent sequelae were reported as 50 (87.7%) patients recovered, 2 (3.5%) patients had persistent sinus node dysfunction as evidenced by subsequent EKG, and 2 (3.5%) patients expired as a result of lithium toxicity.

## Discussion

**Figure 3:**
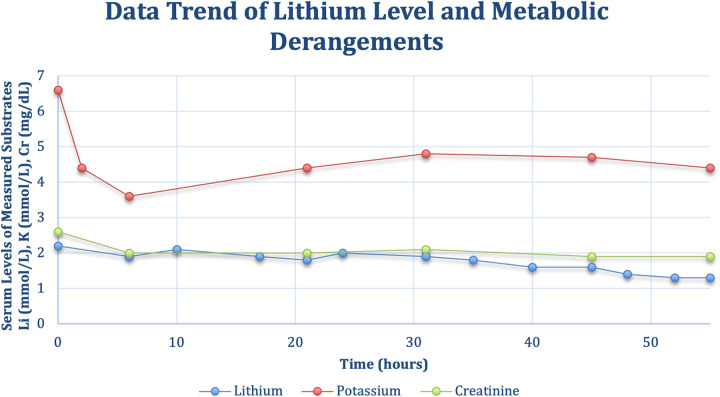
Timeline of monitoring for serial lithium levels potassium, and creatinine as a marker of renal function over the course of the patient's hospital admission.

In this case report and systematic review, we herein demonstrate the first successful reported use of a novel treatment methodology to rapidly correct lithium-induced cardiac toxicity in a patient with long-standing bipolar disorder on chronic lithium treatment while also generating the most up-to-date and comprehensive clinical compendium of lithium-associated sinus node dysfunction (15–17). The administration of theophylline can be understood to be the primary factor attributing to sinus node recovery and improvement in bradycardia in our patient, as a careful analysis of the temporal sequence of events, and the patient's response to interventions strongly supports the primary role of theophylline. At presentation, the patient exhibited both lithium toxicity of 2.2 (mmol/L) and significant hyperkalemia of 6.6 (mmol/L), both of which can contribute to significant bradyarrhythmias and EKG abnormalities. The initial EKG changes, particularly the absence of p-waves could indeed be attributed to a combined effect of such significant electrolyte aberrancies, however the subsequent clinical course provides compelling evidence for the specific efficacy of theophylline in this case. The temporal association between interventions and clinical response is crucial to our interpretation in support for the case of theophylline (Figure 3). Despite initial management, including the administration of atropine and correction of electrolyte imbalances, there was only a transient improvement in the patient's cardiac rhythm with subsequent decline. Notably, even though the patient's potassium levels normalized rapidly, decreasing from 6.6 (mmol/L) to 4.4 (mmol/L) within two hours, and a significant improvement of renal function with a decrease of creatinine from 2.6 (mg/dl) to 2.0 (mg/dl) within 6 h, the patient till experienced a recurrence of junctional bradycardia with absence of p-waves approximately 12 h after admission as evidenced in Figure 1C. This temporal evidence of persistent SND in the setting of corrected metabolic derangements and improved renal function can be most likely attributed to the supratherapeutic levels of lithium. It points to a more direct and lasting effect on sinus node dysfunction which is consistent with the known pharmacodynamics of lithium with accumulation in cardiac tissues. This tissue deposition of lithium can likely continue to affect sinus node function even as serum levels begin to normalize (20).

Furthermore, the administration of theophylline marked a clear turning point in the patient's clinical course. Theophylline is thought to increase intracellular cAMP levels in cardiac tissue through phosphodiesterase inhibition (21), which given the temporal association with sinus node improvement is the likely mechanism of improved sinus node automaticity we observed in this case. Within 12 h of theophylline administration, telemetry capture revealed a progressive shortening of sinus pauses, from an initial arrest of 3.8 s to brief pauses of 1.32 s, culminating in a complete resolution of SND and return to normal sinus rhythm. This rapid and sustained response to theophylline, occurring when both lithium and potassium levels were closer to normal, strongly suggests a direct effect of theophylline on sinus node function consistent with prior reports in the clinical literature establishing the efficacy of theophylline in treating sinus node dysfunction (22, 23).

Symptomatology of acute lithium toxicity is fairly consistent at mild concentrations with presentations of dizziness, tremor, lethargy, nausea and vomiting at moderate to severe concentrations symptoms can progress to confusion, agitation, delirium, tachycardia and hypertonia (15–17). Cardiac symptomatology can present over a wide range of lithium concentrations with exposure ranging from acute to chronic with evidence of QTc prolongation, t-wave flattening, sinus node dysfunction, and life-threatening arrythmias. The mainstay of treatment for acute lithium toxicity is judicious fluid resuscitation, but primarily driven by the degree of symptoms. If the patient demonstrates signs and symptoms of severe lithium poisoning or is having a renal failure due to its small volume of distribution and marginal protein binding, hemodialysis should be considered for rapid elimination (17, 18).

Cardiotoxic effects of lithium intoxication typically resolve with proper elimination, but if severely acute as in the case of profound bradycardia, the use of temporary pacing or permanent pacemaker implantation may be required. In non-lithium-associated sinus node dysfunction, methylxanthines (theophylline and aminophylline) are an accepted treatment choice given their positive chronotropic effects (4). The majority of data supporting the efficacy of these medications has been evaluated primarily in the setting of either spinal cord injury or post-heart transplant patients, although there are reports of successful theophylline use in other etiologies of SND (13, 19). Under the presumptive conditions that transient sinus node dysfunction will resolve with elimination of toxic concentrations of lithium, temporary chronotropic support would be preferable to more invasive measures such as temporary pacing, hemodialysis, or implantation of a permanent pacemaker.

In conclusion, this case highlights the potential of theophylline as an effective treatment for lithium-induced SND, particularly in cases where the dysfunction persists despite correction of other contributing factors. The rapid and sustained response to theophylline observed in this patient, supported by extensive literature on its efficacy in treating sinus node dysfunction, provides a compelling argument for its use in similar clinical scenarios Given the summary of findings regarding patient prognosis as described in our report, it can be postulated that theophylline may be potentially non-inferior to other treatment methodologies albeit this is not represented by comparative statistical analysis. We propose that further studies are needed before theophylline comparing the efficacy of theophylline to other treatment modalities in lithium-induced SND before theophylline can be recommended as a treatment specifically for lithium-induced sinus node dysfunction. Given the findings that our report provides, initial clinical utility for the use of Theophylline in rapid correction of lithium-induced sinus node dysfunction is evident.

## Data Availability

The original contributions presented in the study are included in the article/Supplementary Material, further inquiries can be directed to the corresponding author.
